# Construction of a nomogram to predict urethral stricture after transurethral resection of the prostate: A retrospective cohort study

**DOI:** 10.1371/journal.pone.0313557

**Published:** 2025-02-12

**Authors:** Wan Mokhzani Wan Mokhter, Xiaoping Duan, Jin Yang, Mohamed Ashraf Mohamed Daud

**Affiliations:** 1 School of Medical Sciences, Universiti Sains Malaysia, Health Campus, Kubang Kerian, Kelantan, Malaysia; 2 Department of Urology, Affiliated Hospital of Chengdu University, Chengdu University, Chengdu, China; 3 Department of Surgery, School of Medical Sciences, Universiti Sains Malaysia, Health Campus, Kubang Kerian, Kelantan, Malaysia; University General Hospital of Heraklion, University of Crete, Medical School / Training and Research in Urological Surgery and Technology (T.R.U.S.T) Group, GREECE

## Abstract

**Background:**

To investigate the risk factors for urethral stricture (US) in patients with benign prostatic hyperplasia (BPH) after transurethral resection of the prostate (TURP) and to construct a nomogram model with predictive features.

**Methods:**

Clinical data of 400 patients with BPH who underwent TURP between June 2020 and June 2023 at Chengdu University Hospital were retrospectively collected. The data were divided into US group and no US group. Univariate and multivariate logistic regression analyses were performed sequentially to identify independent risk factors associated with US. Based on the results of the multivariate analysis, a nomogram model predicting the risk of US was constructed. We assessed the discriminatory power and calibration of the models using the C index, ROC curves, and calibration plots. In addition, we performed a decision curve analysis to validate the clinical utility of the model.

**Results:**

Data from a total of 400 patients were included in this study, and 35 (8.75%) were diagnosed with US. The results of univariate and multivariate analyses indicated that the following five factors age, prostate size, Preoperative indwelling catheter, Preoperative urethral dilation, Postoperative indwelling catheter time were independent influences on the risk of US. Nomogram model of US was constructed using these independent influences. The area under the curve (AUC) of the subject’s operating characteristic was 0.916 (95% CI: 0.868–0.959), and after internal validation, the corrected C-index remained at 0.916. This further validates the accuracy and reliability of the predictive model. Calibration plots and decision curve analyses demonstrated the good clinical value of the column-line diagram model.

**Conclusions:**

The nomogram model we constructed can have some guidance in clinical work.

## Introduction

BPH is the most common urinary disorder among middle-aged and older men worldwide [1]. Currently, surgery is one of the most effective treatments for BPH [2]. With the development and progress of medicine, various new technologies continue to emerge and are applied in the treatment of BPH. However, TURP Remains One of the Standard Minimally Invasive Methods for Treating BPH [3, 4]. TURP is a minimally invasive procedure that removes prostate tissue through the urethra without an external incision [5, 6]. TURP typically results in a shorter hospital stay, faster recovery, and less post-operative pain than open prostatectomy. In addition, TURP is often more cost-effective than newer, more technologically advanced procedures such as robotic-assisted surgery [7, 8].

Urethral stricture (US) is one of the major late complications after TURP [9]. Through a literature review, we learnt that there are several explanations for the occurrence of US after TURP. However, the exact etiology of this complication remains controversial. Studies have shown that possible causes of US formation after TURP include infection, mechanical trauma, and long-term catheter retention [10, 11]. Several studies have also shown that the increased incidence of US after TURP is mainly related to the energy used, the calibre of the instruments, and the time of insertion of the postoperative catheter [12]. Recent studies have shown that catheter retention time, length of hospital stay, and bladder irrigation time are major risk factors for the development of postoperative US [13].

The aim of this study was to develop and validate a personalized column line drawing model that accurately predicts the risk of postoperative US in patients with BPH treated with TURP. High-risk individuals can be identified based on various indicators measured at admission, allowing timely preventive interventions. This innovative precision medicine approach demonstrates significant clinical utility and has the potential to substantially reduce the incidence of postoperative US in patients.

## Methods

### Patients

This study followed the STROCS guidelines and conformed to the principles of the 1964 Declaration of Helsinki and its subsequent amendments. This study was approved by the Institutional Review Board of The Affiliated Hospital of Chengdu University on 10/04/2023, which adopted the submitted application form for waiver of informed consent or waiver of some elements of informed consent.

We retrospectively collected clinical data on 12/04/2023 from 540 patients with BPH who were treated with TURP between June 2020 and June 2023 at The Affiliated Hospital of Chengdu University. And all data were anonymized by professional staff in the case room before access.

The inclusion criteria for this study were:

1. age ≥65 years
2. Meets the indications for TURP surgery
3. BPH diagnosed by histology

The following exclusion criteria were applied:

1. patients who did not receive surgical treatment
2. patients with incomplete clinical data
3. Preoperative diagnosis of US
4. Postoperative pathological examination for prostate cancer and other diagnoses
5. Previous prostate or other transurethral related surgery.
6. Urethral stricture due to neurogenic bladder outlet obstruction confirmed by urodynamics

Based on the exclusion criteria, a total of 140 patients were excluded, resulting in a retrospective cohort study involving 400 patients. The mean follow-up in this study was 6 months, ranging from a minimum of 3 months to a maximum of 9 months. Patients were categorized into two groups based on whether or not urethral strictures occurred postoperatively. The screening process and participant flow are illustrated in a flow diagram (S1 Fig).

### TURP

The surgery was performed by two board certified urologists who had attended and passed their residency training and had the appropriate number of years of surgical experience and title. The trainees participated in the surgical learning but were not responsible for the primary operations. All procedures were performed using the same electrosurgical scope, scalpel, and irrigation system with a 26 V energy OLYMPUS bipolar electrosurgical endoscope, and continuous irrigation was used during the procedure. The type of anesthesia was combined lumbar-hard anesthesia with uniform 0.5% bupivacaine in all patients. Postoperatively, all tissue blocks are cleaned and sent for pathologic examination. An F22 triple lumen catheter is then placed to continuously flush the bladder.

### Diagnosis of US

Initially, based on the patient’s history of rehospitalization, medical records, physical examination, and clinical presentation, a preliminary assessment is made to determine the possibility of urethral stricture in the patient [14]. Subsequently, the next step involves measuring the patient’s maximum urinary flow rate. A decrease in the maximum urinary flow rate further increases the likelihood of US occurrence [15]. Finally, retrograde urethrogram can be used to accurately assess the occurrence, location, length, and associated abnormalities of US [16]. Some of the patients’ urethral strictures are diagnosed through outpatient urethral ultrasound examinations [17]. This study rigorously followed the aforementioned steps to confirm the occurrence of urethral stricture in patients with BPH after TURP. In this study, a maximum urinary flow rate measured by uroflowmetry of <10 mL/s was considered indicative of US [18]. In order to avoid misdiagnosis of bladder neck contracture, urethrocystoscopy and retrograde urethrogram were performed to confirm US. The urethrogram results showed that among the 35 patients with urethral strictures, 21 (60.0%) had anterior urethral strictures and 14 (40.0%) had posterior urethral strictures. The length of the stricture ranged from 0.4 to 4.1 centimeters, with 21 (60.0%) less than 1.0 centimeter, 11 (31.4%) between 1.0 and 3.0 centimeters, and 3 (8.6%) greater than 3.0 centimeters.

### Data collection

We collected data on 20 potential risk factors for US by reviewing relevant literature and clinical records of patients in our institution. Demographic factors included age, smoking, and alcohol use. The comorbidities considered were hypertension, diabetes, and chronic kidney disease. Operation variables are divided into prostate size, duration of BPH, surgery duration, preoperative indwelling catheter, preoperative urinary tract infection, preoperative urethral dilation, preoperative urinary retention, intraoperative blood loss, postoperative continuous bladder irrigation time, and postoperative indwelling catheter time. Laboratory parameters obtained within 24 hours of admission included white blood cell count, hemoglobin level, blood urea nitrogen, and creatinine. Prostate values including anterior and posterior diameters, upper and lower diameters and weight values were measured by transrectal color ultrasound in all patients. The data retrieval of electronic medical records for all urology patients was conducted by two researchers from the Affiliated Hospital of Chengdu University. They both received specialized training in data collection.

### Statistical analysis

Categorical variables were expressed as numbers combined with percentages (%) and then compared by chi-square test. Normal distribution of continuous variables was tested by Shapiro-Wilk test. Normally distributed continuous data were expressed as mean ± standard deviation and then the results were analysed using independent samples t-test. Non-normally distributed continuous data were expressed as median (interquartile spacing) and compared using the Mann-Whitney U test.

First, a χ^2^ test was performed on all factors of the included patients to screen for potential influences, and the level of significance was set at P<0.05. Variables that were significant were included in subsequent analyses. In order to assess the relationship between various potential risk factors and US, univariate logistic regression analyses were conducted for each factor. In univariate analyses, variables with a significance level of P<0.05 were included in multivariate logistic regression analyses for initial prediction of possible risk factors. The variance inflation factor was used to assess multicollinearity in the multivariate model. Based on the multivariate analysis, a nomogram for predicting the risk of urethral stricture was developed using R software in the training set.

To assess the performance of the predictive column-line plots, we plotted the subject operating characteristic curves and calculated the area under the curve to assess the sensitivity and specificity of the model. Calibration plots were generated to examine the accuracy of the nomogram. The predictive model’s clinical utility was assessed using decision curve analysis to determine if it improved forecasted net income. All clinical data were statistically analysed using SPSS version 26.0 (IBM USA), and then column-line plots were constructed using R version 4.0.3 (R Foundation for Statistical Computing, USA) based on the results of the independent influence factors.

## Results

### Baseline clinical and demographic characteristics of patients

According to the inclusion and exclusion criteria, a total of 400 patients with BPH treated by TURP were included in this study. As depicted in the study design flowchart, there were 35 patients in the US group (8.75%) and 365 patients in the non-US group (91.25%). As shown in S1 Table, the demographic and baseline clinical characteristics of the two groups of patients were comparable. Baseline characteristics of patients with and without US are also presented separately.

### Independent risk factors in the training set

As shown in Table 1. Univariate analysis revealed several risk factors for US in patients with BPH after TURP. These factors included age, diabetes, prostate size, preoperative indwelling catheter, preoperative urinary tract infection, preoperative urethral dilation, intraoperative blood loss, operative time, postoperative indwelling catheter time, and hemoglobin concentration level. The above risk factors were included in multivariate logistic regression analyses. Age, prostate size, preoperative indwelling catheter, preoperative urethral dilation, and postoperative indwelling catheter time are the ultimate independent factors. Age(OR:1.121; 95%CI:1.044~1.204; p-value: 0.002); Prostate size(OR: 1.038; 95%CI: 1.014–1.062; p-value: 0.002);Preoperative indwelling catheter(OR: 5.413; 95%CI: 1.872–15.655; p-value: 0.002); Preoperative urethral dilation(OR: 0.049; 95%CI: 0.011–0.220; p-value<0.001);Postoperative indwelling catheter time(OR: 2.147; 95%CI: 1.405–3.280; p-value<0.001); Furthermore, a predictive formula was created based on regression coefficients and constants: logit(p) = -17.751 + 1.059 * age + 1.717 * prostate size—1.095 * preoperative urethral dilation + 1.106 * preoperative indwelling catheter + 1.124 * postoperative urinary catheter time. The outcomes of the multivariate logistic regression analysis, including the intercept, β coefficient, and odds ratio, are presented in Table 2.

**Table 1:** Univariate and multivariate analysis of US.

Characteristics	Univariate			Multivariate		
	OR	95%CI	P-value	OR	95%CI	P-value
Age	1.121	1.066–1.179	<0.001	1.121	1.044–1.2044	0.002
Smoking	0.513	0.296–1.837	0.513	NA	NA	NA
Alcohol	0.981	0.364–2.642	0.970	NA	NA	NA
Hypertension	1.318	0.657–2.644	0.437	NA	NA	NA
Diabetes	2.893	1.265–6.620	0.012	1.880	0.519–6.812	0.928
Chronic kidney disease	2.350	0.752–7.341	0.142	NA	NA	NA
Prostate size	1.041	1.026–1.057	<0.001	1.038	1.014–1.062	0.002
Preoperative indwelling catheter	4.801	2.341–9.847	<0.001	5.413	1.872–15.655	0.002
Duration of BPH	1.037	0.886–1.212	0.653	NA	NA	NA
Preoperative urinary tract infection	2.364	1.137–4.915	0.021	1.794	0.561–5.737	0.325
Intraoperative blood loss	1.027	1.012–1.042	<0.001	0.993	0.960–1.027	0.673
preoperative urethral dilation	0.328	0.113–0.953	0.041	0.049	0.011–0.220	<0.001
Preoperative urinary retention	1.364	0.654–2.843	0.408	NA	NA	NA
Operative time	15.519	5.330–45.188	<0.001	3.399	0.329–35.125	0.304
Postoperative continuous bladder irrigation time	0.824	0.347–1.952	0.824	NA	NA	NA
Postoperative indwelling catheter time	2.590	1.948–3.444	<0.001	2.147	1.405–3.280	<0.001
WBC count	1.062	0.953–1.185	0.277	NA	NA	NA
HGB level	0.982	0.964–1.000	0.048	0.988	0.962–1.015	0.396
BUN	1.022	0.938–1.114	0.614	NA	NA	NA
Cr	1.001	0.997–1.004	0.791	NA	NA	NA

Abbreviations: US, urethral stricture; BPH, benign prostatic hyperplasia; WBC, white blood cell; HGB, hemoglobin; BUN, blood urea nitrogen; Cr, creatinine

**Table 2:** Multivariate analysis of US after TURP.

Risk factor	β	S E	Wald	OR	95%CI	p-value
Age	0.114	0.036	9.922	1.121	1.044–1.204	0.002
Prostate size	0.037	0.012	10.048	1.038	1.014–1.062	0.002
Preoperative indwelling catheter	1.689	0.542	9.717	5.413	1.872–15.655	0.002
Intraoperative urethrotomy	-3.016	0.766	15.502	0.049	0.011–0.220	<0.001
Postoperative indwelling catheter time	0.764	0.014	0.722	2.147	1.405–3.280	<0.001

β, beta; SE, standard error; Wald: Wald statistic; OR, odds ratio; CI, confidence interval; The p-value is used to determine whether the relationship between each independent variable and US is statistically significant.

### Nomogram model establishment

**Fig 1:**
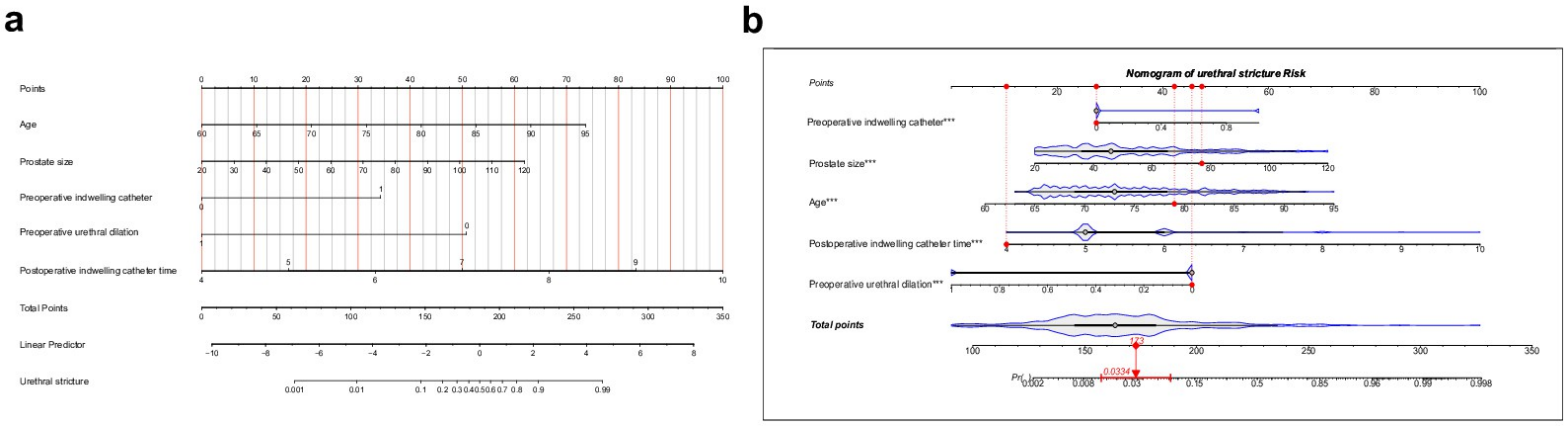
Nomogram for predicting US after transurethral resection of the prostate. (a) Five variables were included in the nomogram prediction model, age, prostate size, preoperative indwelling catheter, preoperative urethral dilation and postoperative indwelling catheter time. (b) Dynamic nomogram as an example.

Based on a multivariate logistic regression analysis, we developed an individualized nomogram model to predict US risk in BPH patients. The nomogram model consists of 5 independent US risk factors: age, prostate size, preoperative indwelling catheter, preoperative urethral dilation and postoperative indwelling catheter time, which are depicted in Fig 1. Each factor in the nomogram is assigned a corresponding score, and the total score reflects the predicted US risk. For example, a 70-year-old male patient (25 points), prostate size 60 (38 points), without preoperative indwelling catheter (28 points), without preoperative urethral dilation (46) and a urinary catheter for 8 days (70 points) would have a total score of 207 points, corresponding to a predicted US risk of 73%.

### Nomogram model validation

**Fig 2:**
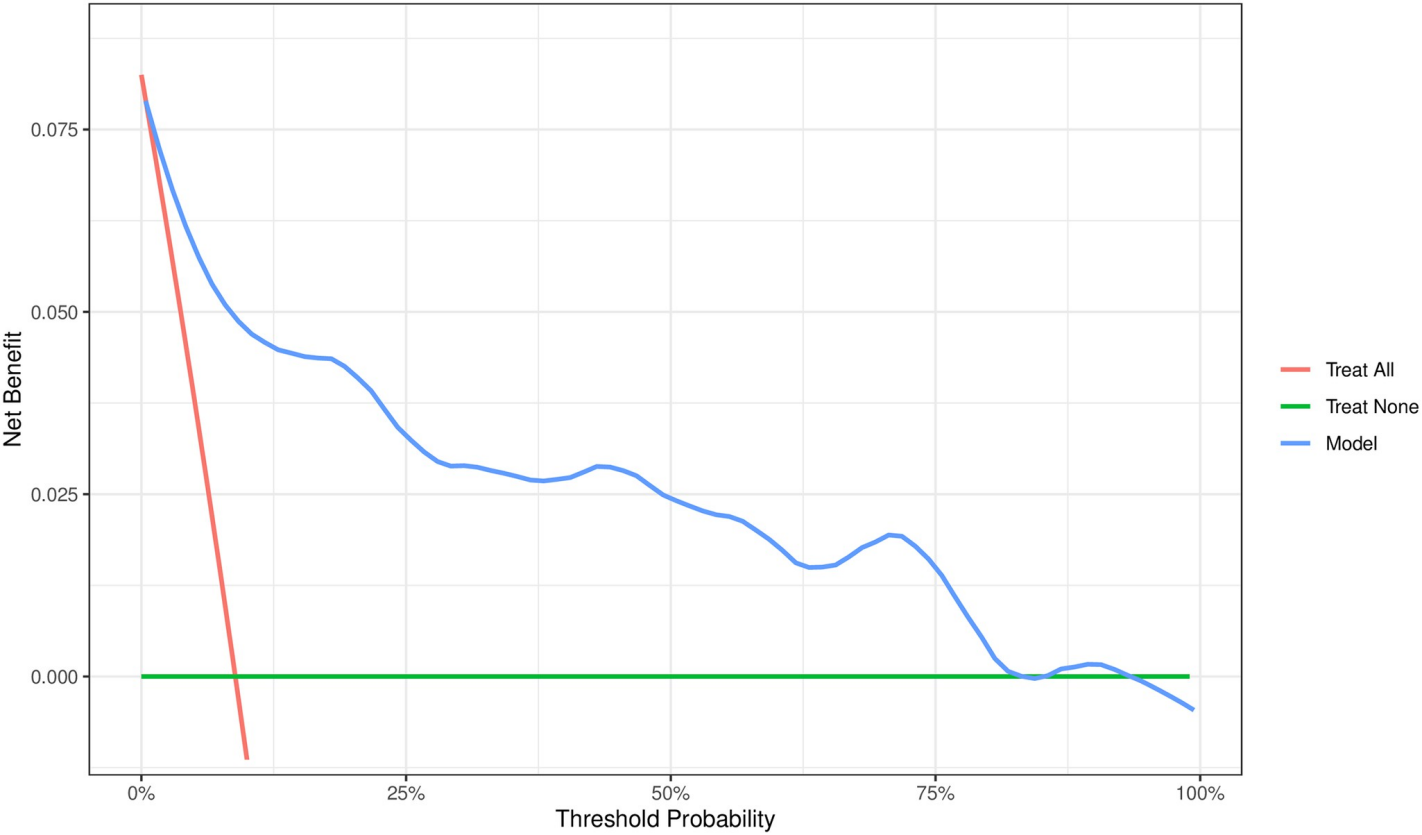
Decision curve analysis of the nomogram. The blue line indicates the net benefit of our model. The red line assumes that all patients develop urethral stricture. The green line assumes that no patient develops urethral stricture.

After establishing the model, model internal validation was conducted through repeated resampling (1000 times) and a calibration curve was plotted to assess the model’s goodness of fit (S2 Fig). The diagnosis for collinearity indicated that the variance inflation factors for these risk factors ranged from 1.059 to 1.171, signifying the absence of multicollinearity. The calibration curves showed that the actual probability and predicted probability intersection curves floated around the ideal curve after repeated sampling for in-model validation, which indicated that the predictive model was well fitted. The Hosmer-Lemeshow test (X-squared = 6.6332, df = 8, p-value = 0.5767) indicates that the regression model fits well, suggesting good predictive performance and model adequacy. Receiver operating characteristic curve analysis results show that the area under the curve (AUC) for predicting post-TURP urethral stricture nomination is 0.916 (95% CI: 0.868–0.959), indicating excellent discriminatory ability (S3 Fig). Decision curve analysis evaluates the expected net benefit for each patient. According to the decision curve, the model generates net benefit within the threshold probability range of 2.25% to 83.00%, as illustrated in Fig 2. S4 Fig illustrates the ROC curves for the five independent risk factors.

## Discussion

US is the most common late complication following TURP in patients with BPH. It significantly affects patients’ prognosis and recovery, causing considerable inconvenience. Studies have found that symptoms of US develop in 4.5–13% of patients after TURP [19]. According to Kulkarni’s study, the incidence rate of US following TURP ranges from 2.2% to 9.8%. In this study, the incidence rate of US is 8.75%, falling within this range. US is classified into anterior US and posterior US, with anterior US accounting for 92.2%. Studies indicate that the primary site of US occurrence is the bulbar urethra. In our study, 60% of patients (21/35) exhibit bulbar US. It typically occurs several weeks to months after transurethral prostatectomy. Clinical manifestations include decreased urine stream diameter, split stream, prolonged urination time, and urinary retention [20, 21]. The occurrence of US leads to prolonged hospitalization, resulting in greater financial burden for patients and significant inconvenience in their daily lives. Therefore, early identification and intervention of US are crucial for these patients.

In order to investigate the predictive rate of US occurrence, we conducted a retrospective study involving a large number of elderly patients with benign prostatic hyperplasia. Utilizing collected clinical data, we formulated a nomogram comprising multiple clinical and laboratory parameters reflecting key factors associated with US incidence. Through statistical analysis and multivariate logistic regression, we determined the weighted coefficients of five variables: age, prostate size, preoperative indwelling catheter, preoperative urethral dilation, and duration of catheterization. By incorporating these variables and coefficients, we developed a simplified, intuitive nomogram-based risk assessment tool. Internal and external validation of the nomogram demonstrated good accuracy and stability in predicting urethral stricture. Therefore, this model can provide clinicians with valuable clinical assistance in reducing the incidence of US.

### Age

As patients age, the elasticity and structural integrity of the urethra may change, making them more susceptible to strictures. In addition, older patients may have more comorbidities and a decreased ability to heal tissues, all of which increase the risk of postoperative urethral strictures. There is a positive correlation between prostate size with age in patients with BPH. Prostate size has been reported to have an independent effect on US after TURP, and therefore age is considered to be one of the potential risk factors for urethral stricture after TURP [12, 22]. In conclusion, while some studies suggest that age may be a potential risk factor for postoperative US, current data are not conclusive. Other factors such as surgical technique and patient characteristics may also influence the risk of US. We need to conduct further research to establish whether there is a relationship between patient age and the occurrence of postoperative US.

### Prostate size

The larger the preoperative prostate volume of the patient, the more frequent the intraoperative urethral endoscopic procedures, resulting in a longer surgical duration. Repeated manipulation and rotation of the electroscope during the procedure can cause damage to the urethral membrane, especially in patients with relatively small urethral openings, which in turn leads to a higher likelihood of urethral stricture [23]. The larger size of the prostate causes mechanical compression and obstruction of the urethra. This compression can further damage the urethral tissue. In addition, a larger prostate may block the urine flow more severely, increasing the risk of postoperative US [24]. However, existing research has shown that preoperative prostate volume is associated with the occurrence of bladder neck contracture following TURP, but it does not affect the occurrence of postoperative US [25].

### Preoperative indwelling catheter

There is a correlation between preoperative indwelling catheters and the development of US. Urethral stricture is caused by injury to the urethra and can lead to urethral obstruction. It further leads to complications such as urinary incontinence, urinary tract infections and renal insufficiency. This is because the use of catheters may cause damage to the urethra and may also lead to the development of urethritis, which are both factors that directly influence the development of urethral strictures [26]. The size of the catheter used during preoperative catheterisation may lead to urethral strictures. Larger catheters may cause more trauma to the urethra during insertion or removal, which may lead to scarring and stricture formation. The material of the catheter, such as latex or silicone, may also impact the development of strictures. Some materials may be more biocompatible and less likely to cause irritation or inflammation in the urethra, reducing the risk of strictures [27]. However, it has been suggested that the material and size of the urethral catheter have no significant effect on the formation of US [28]. Some studies have suggested that preoperative indwelling catheters prevent postoperative US [29].

### Preoperative urethral dilation

Preoperative urethral dilatation was a protective factor for There are reports validating prostate size as an independent influence on post-TURP urethral stricture after TURP in this study. Urethral dilatation is a procedure in which the internal diameter of the urethra is dilated or maintained by means of an instrument such as a urethral probe to ensure smooth urination. This procedure is often used to treat urethral strictures and improve urinary symptoms [30]. On the one hand, the width of the urethra is increased by mechanical dilatation of the urethral probe, and on the other hand, the urethral mucosa is massaged by urethral dilatation, which results in improvement of local blood circulation and softening of periurethral scarring. These are beneficial in reducing the risk of postoperative urethral stricture. Some studies have also shown that preoperative urethral dilatation reduces the incidence of US [31].

### Catheter indwelling time

Prolonged catheter retention exerts pressure on the urethral mucosa and surrounding tissues, impacting local blood circulation. To prevent postoperative bleeding, some surgeons remove the catheter to achieve hemostasis. They may also use indwelling catheters to pull and compress the urethra, hindering drainage of infected fluid from the urethral mucosa to the ureter. This exacerbates local tissue damage, resulting in ischemia and necrosis of the urethral mucosa, potentially causing urethral stricture. Local tissue damage further aggravates the urethral mucosa, leading to ischemia and necrosis, which can easily induce urethral stricture [32]. Studies have indicated that postoperative use of COX-2 inhibitors can effectively prevent the occurrence of urethral stricture. It achieves effective prevention through specific interference with scar formation [33]. Postoperative reasonable choice of catheter, strict control of catheter retention time, and regular flushing catheter, do a good job of skin cleaning of the urinary orifice, regular replacement of the urine bag, to avoid the invasion of pathogenic bacteria in the urethra; after the removal of the catheter, to guide the patient. After removal of the catheter, the patient should be instructed to perform pelvic floor muscle exercise to prevent urethral stenosis. Prolonged indwelling urinary catheter will produce continuous stimulation of the urethral mucosa and lead to decreased resistance to pathogenic bacteria, increasing the risk of urethral infection, leading to the inability of smooth drainage of the urethra, and then increasing the risk of damage to the mucosa of the urethra necrotic urethral oedema, urethral ulceration, and urethral stenosis. Therefore, if possible, remove the urinary catheter as early as possible, and instruct the patient to drink water reasonably after removing the urinary catheter to promote urine production and restore self-urination, so as to reduce the risk of US as much as possible.

## Limitations

Nomograms are increasingly used as a novel non-invasive visualization tool for clinical predictive modelling. In our study, we have successfully built and validated a nomogram model. The nomogram was derived from a training cohort and validated within the institution, which showed good discrimination and calibration. However, internal validation may also overestimate model discrimination. Unlike traditional models focused on laboratory parameters, this nomogram incorporates routine clinical data that can be easily obtained upon presentation. By analysing patient data through nomogram, clinicians can stratify patients on admission and direct targeted preventive interventions for high-risk patients. Consequently, this model serves as an effective clinical decision aid to facilitate precision prevention of There are reports validating prostate size as an independent influence on post-TURP urethral stricture within this vulnerable population.

However, We also recognise the limitations of this study. First, this study was conducted in only one specific hospital., which may limit the applicability of our findings to patients in other healthcare facilities. Secondly, the retrospective design introduces a potential for bias and restricts the ability to establish causal relationships between the identified hazard factors and the occurrence of urethral stricture. Thirdly, although the nomogram model displayed good discrimination and predictive performance, it was solely validated within the derivation cohort utilized for its development. Consequently, external validation using independent datasets is necessary to confirm the generalizability of the model. Fourthly, the nomogram only evaluates patients treated with TURP and may not be applicable to patients with BPH treated with other minimally invasive procedures. In addition, decision curve analyses suggest that nomograms may be beneficial to patients. However, the actual clinical utility in improving treatment decisions and patient outcomes requires further evaluation.

## Conclusion

We developed and validated nomograms to predict the risk of post-TURP urethral stricture in this specific population of BPH patients. The aim was to assess the practicality of these nomograms. The nomogram demonstrated strong discriminatory and calibration capabilities, demonstrating its reliability in risk prediction. The tool enables early identification of high-risk patients, leading to targeted preventive interventions. By implementing the nomogram, we can effectively reduce the occurrence of urethral strictures and associated complications.

## Supporting information

S1 Fig Flow diagram of study design. (TIF)

S2 Fig Internal validation calibration curve plot. (TIF)

S3 Fig Receiver operating characteristic curve. (TIF)

S4 Fig The ROC curve of five independent risk factors. (TIF)

S1 Table Baseline characteristics of the patients between US and non-US groups. (DOCX)

## Abbreviations

BPH: benign prostatic hyperplasia
ROC: Receiver operating characteristic curve
TURP: Transurethral resection of the prostate
US: urethral stricture
