# Supplementary figures and images for: Construction of a nomogram to predict urethral stricture after transurethral resection of the prostate: A retrospective cohort study
Source: PLoS One. 2025 Feb 12;20(2):e0313557. doi: 10.1371/journal.pone.0313557 (PMC11819526; doi:10.1371/journal.pone.0313557)

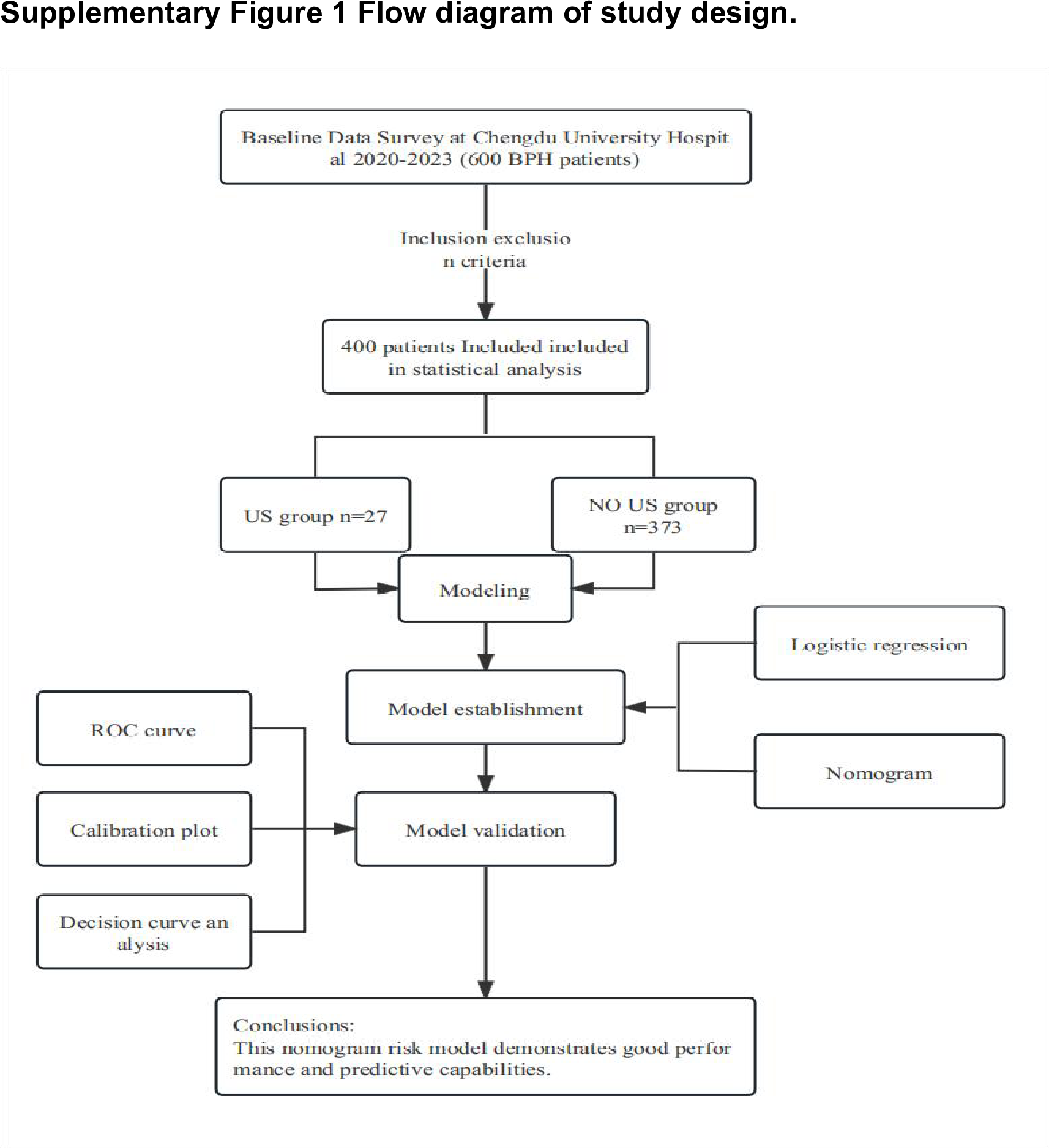

Supplement: S1 Fig — (TIF) [file pone.0313557.s001.tif]

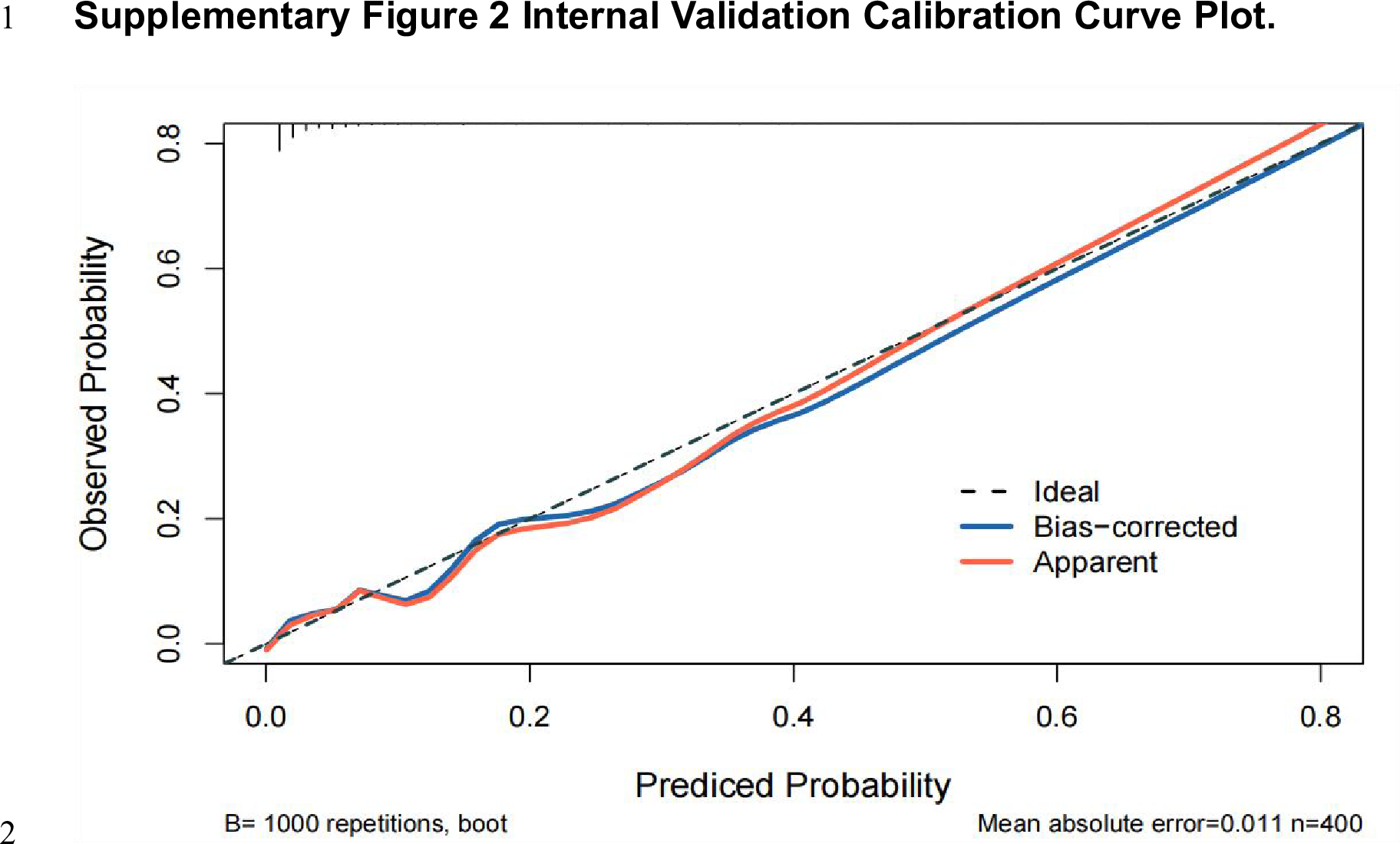

Supplement: S2 Fig — (TIF) [file pone.0313557.s002.tif]

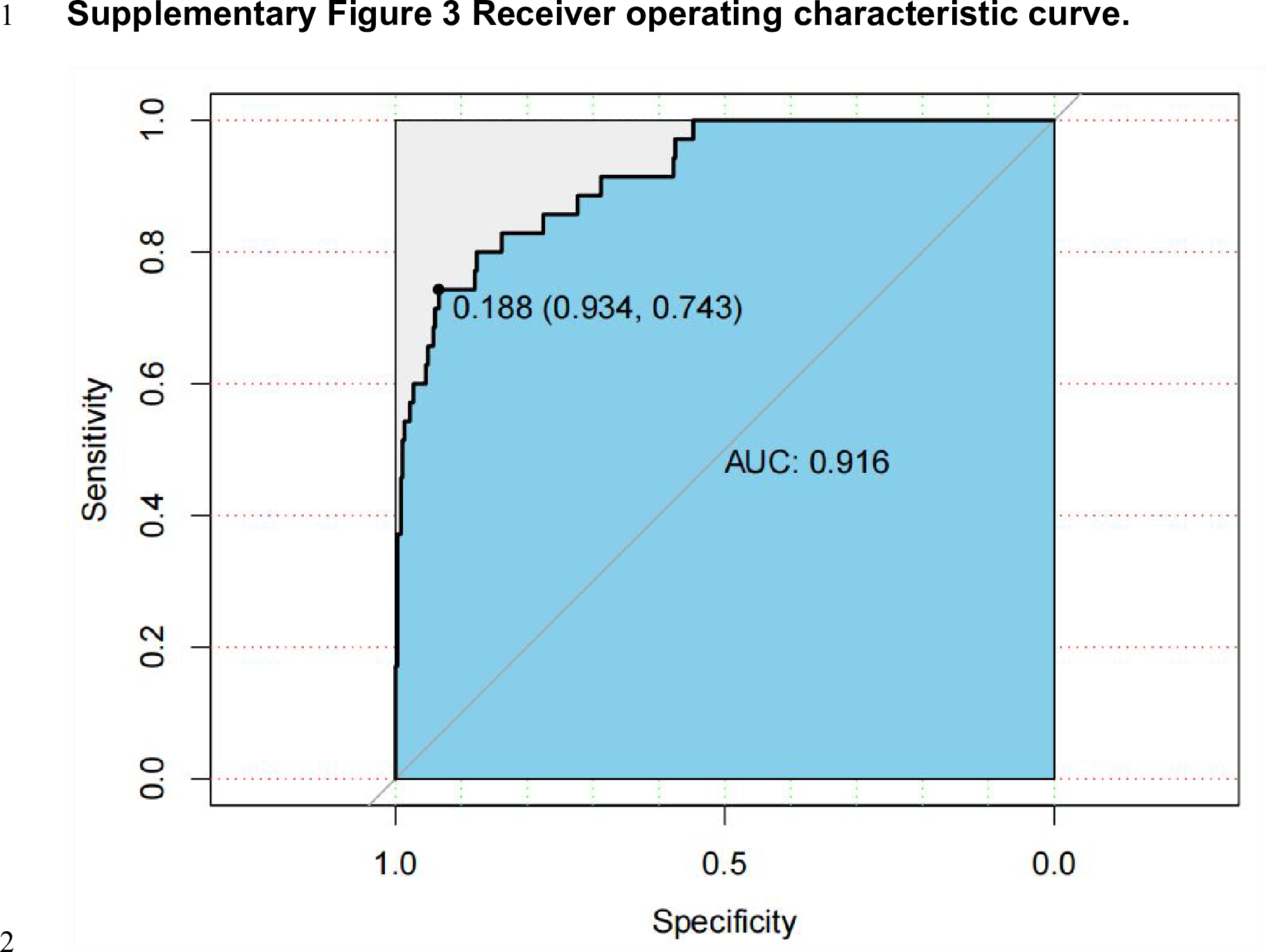

Supplement: S3 Fig — (TIF) [file pone.0313557.s003.tif]

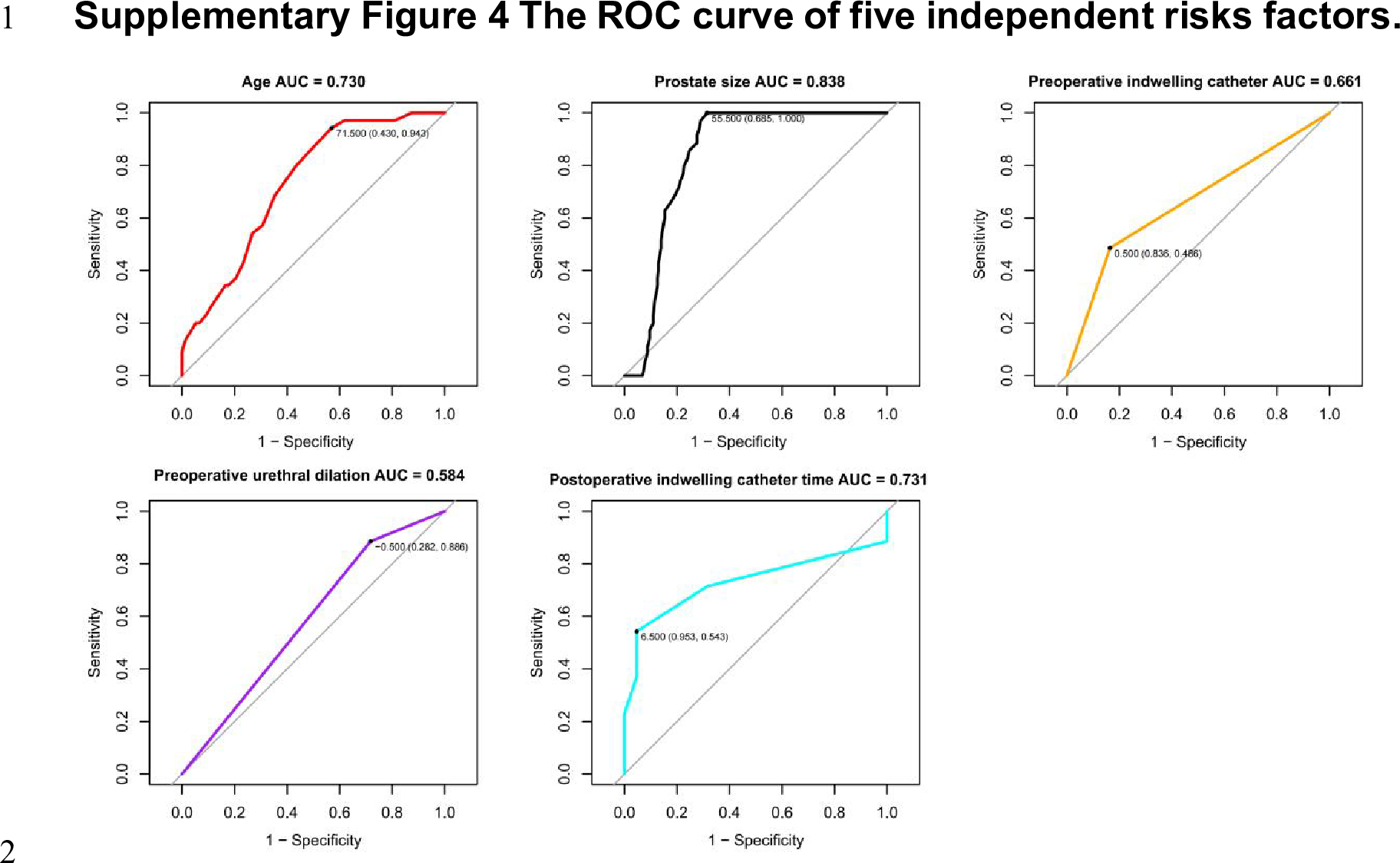

Supplement: S4 Fig — (TIF) [file pone.0313557.s004.tif]
