# Supplementary material for: Construction of a nomogram to predict urethral stricture after transurethral resection of the prostate: A retrospective cohort study
Source: PLoS One. 2025 Feb 12;20(2):e0313557. doi: 10.1371/journal.pone.0313557 (PMC11819526; doi:10.1371/journal.pone.0313557)
Supplement: S1 Table — (DOCX) [file pone.0313557.s005.docx]

**Supplementary Table1 Baseline characteristics of the patients between US and non-US groups.**

| Variables | Total patients  (n = 400) | Groups | | †p for Trend |
| --- | --- | --- | --- | --- |
|  |  | Without US  (n = 365) | US  (n = 35) |  |
| Demographic |  |  |  |  |
| Age, × year (Mean±SD) | 74.31(6.70) | 73.82(6.49) | 79.40(6.84) | <0.001 |
| Smoking (n, %) | 86(21.5) | 80(21.9) | 6(17.1) | 0.511 |
| Alcohol (n, %) | 58(14.5) | 53(14.5) | 5(14.3) | 0.970 |
| Comorbidities |  |  |  |  |
| Hypertension (n, %) | 192(48.0) | 173(47.4) | 19(54.3) | 0.436 |
| Diabetes (n, %) | 48(12.0) | 39(10.7) | 9(25.7) | 0.009 |
| Chronic kidney disease (n, %) | 23(5.8) | 19(5.4) | 4(11.4) | 0.131 |
| Operation |  |  |  |  |
| Prostate size, ×g/L (Mean±SD) | 51.73(22.50) | 49.63(22.27) | 73.57(9.56) | <0.001 |
| Preoperative indwelling catheter, (n, %) | 77(19.3) | 60(16.4) | 17(48.6) | <0.001 |
| Duration of BPH, ×year (Mean±SD) | 2.42(2.09) | 2.41(2.07) | 2.57(2.28) | 0.654 |
| Preoperative urinary tract infection (n, %) | 86(21.5) | 73(20.0) | 13(37.1) | 0.018 |
| Intraoperative blood loss, ×ml (Mean±SD) | 40.00(20.30) | 38.82(20.50) | 52.34(12.88) | <0.001 |
| Intraoperative urethrotomy (n, %) | 107(26.8) | 103(28.2) | 4(11.4) | 0.032 |
| Preoperative urinary retention (n, %) | 113(28.2) | 101(27.7) | 12(34.3) | 0.406 |
| Operative time, × minute (Mean±SD) | 57.69(17.09) | 56.23(16.86) | 72.86(11.22) | <0.001 |
| Postoperative continuous bladder irrigation time, ×minute (Mean±SD) | 1.23(0.42) | 1.23(0.42) | 1.20(0.41) | 0.660 |
| Postoperative indwelling catheter time, ×day (Mean±SD) | 5.54(0.98) | 5.41(0.72) | 6.86(1.90) | <0.001 |
| Laboratory findings |  |  |  |  |
| WBC count, ×10^9/L (Mean±SD) | 9.03(2.84) | 8.99(2.91) | 9.53(1.82) | 0.276 |
| HGB level, ×g/L (Mean±SD) | 122.13(19.21) | 122.72(19.79) | 115.97(9.90) | 0.047 |
| BUN, ×mmol/L (Mean±SD) | 7..14(3.65) | 7.11(3.76) | 7.44(2.19) | 0.615 |
| Cr, ×umol/L (Mean±SD) | 75.15(80.55) | 74.82(84.24) | 78.60(12.46) | 0.791 |

^†^p values are from Fisher's exact test for continuous variables and from the chi-square test for categorical variables.

Abbreviations: US, urethral stricture; SD, Standard deviation; BPH, benign prostatic hyperplasia; WBC, white blood cell; HGB, hemoglobin; BUN, blood urea nitrogen; Cr, creatinine
